# microRNA-32 inhibits the proliferation and invasion of the SGC-7901 gastric cancer cell line *in vitro*

**DOI:** 10.3892/ol.2013.1667

**Published:** 2013-11-07

**Authors:** JIANFENG ZHANG, XIAOLING KUAI, MENGJIAO SONG, XIAOQI CHEN, ZHIHUA YU, HONG ZHANG, ZHENBIAO MAO

**Affiliations:** Department of Gastroenterology, Affiliated Hospital of Nantong University, Nantong, Jiangsu 226001, P.R. China

**Keywords:** gastric carcinoma, cell proliferation, cell movement, microRNA-32

## Abstract

microRNAs (miRNAs) are a class of endogenously expressed, small non-coding RNAs, which suppress their target mRNAs at the post-transcriptional level. miRNAs play key roles in tumor metastasis. The aim of the present study was to investigate the expression of miRNA-32 (miR-32) on the biological behavior of the human gastric cancer cell line, SGC-7901. SGC-7901 cells were transfected with miR-32-mimic, miR-32-inhibitor and empty plasmid vectors using Lipofectamine™ 2000. The expression of GFP was observed by fluorescent microscopy and miR-32 gene expression was detected by quantitative polymerase chain reaction. The cell counting kit-8 assay was performed to evaluate the effect of miR-32 expression on cell proliferation *in vitro*. Alterations in the migration and metastatic potential of SGC-7901 cells, prior to and following miR-32 gene transfection, were assayed by cell chemotactic migration and invasion tests. The results of the current study showed that the proliferation rate of the transfected SGC-7901 cells overexpressing miR-32 is reduced and cell chemotactic migration and invasion potentials is markedly reduced following miR-32-mimic transfection (P<0.05). In addition, the results demonstrated that overexpression of miR-32 greatly inhibits the proliferation and decreases the migration and invasion capabilities of SGC-7901 cells *in vitro*.

## Introduction

Gastric cancer (GC) is one of the most common types of gastrointestinal malignancy worldwide and is the second leading cause of cancer-related mortality (1–3). The majority of patients are diagnosed at an advanced clinical stage, however, the molecular mechanisms are not fully understood.

microRNAs (miRNAs) are a class of small endogenous non-coding RNAs consisting of 19–24 nt, which have high evolutionary conservation. miRNA are important in organism growth, development and the incidence of disease by base pairing to complementary sites in the target mRNA 3′-untranslated region. Thus, miRNA may inhibit the translation or degradation of target mRNA (4–7). Previous studies have indicated that aberrant expression of miRNAs contributes to the initiation and progression of human malignancies, including colon cancer, GC and breast cancer (8,9). However, to date, no specific studies have been conducted to investigate the correlation between miR-32 and GC. The present study investigated the effect of miR-32 on the biological behaviors of the human gastric carcinoma cell line, SGC-7901.

## Materials and methods

### Cell culture and transfection

The human GC cell line, SGC-7901, was obtained from the Cell Bank of the Chinese Academy of Sciences (Shanghai, China) and cultured in Dulbecco’s Modified Eagle’s Medium (Sigma-Aldrich, St. Louis, MO, USA) containing 10% fetal bovine serum, at 37°C in a humidified 5% CO_2_ incubator. Throughout the experiment, cells were used in the logarithmic phase of growth. Transfection was performed using Lipofectamine™ 2000 (Invitrogen Life Technologies, Carlsbad, CA, USA) according to the manufacturer’s instructions. On the day prior to transfection, cells were seeded onto 6-well plates (5×10^5^ cells/well) and inoculated in complete medium without antibiotics (2 ml/well). At 85% confluence, the plasmid DNA (4.0 μg/well) and transfection reagent (10 μl/well) were diluted with RPMI-1640 (250 μl) and stood at room temperature for 5 min. The transfection reagent and dilution of the plasmid DNA were then mixed and stood at room temperature for 30 min. Next, the transfection complexes were added onto 6-well plates (500 μl/well) and cultured at 37°C in a humidified 5% CO_2_ incubator for 48 h. This study was approved by the ethics committee of the Affiliated Hospital of Nantong University (Nantong, China).

### Quantitative polymerase chain reaction (qPCR)

Total RNA was extracted according to the manufacturer’s instructions and dissolved in DEPC water (40 μg). The RNA concentration and purity were detected by ultraviolet-visible spectrophotometer. The specimens, which had A260/A280 values fluctuating between 1.8–2.0, were used for reverse transcription. The process of reverse transcription cDNA synthesis requires the use of RNAse-free centrifuge tubes and must be performed on ice according to the following reaction system: 4 μl 5X reaction buffer, 2 μl dNTP MIX (10 mmol/l), 1 μl RiboLock™ RNase inhibitor, 1 μl RevertAid M-MuLV reverse transcriptase (all from Fermentas Canada Co., Ltd., Burlington, ON, Canada) and RT primer (Sangon Biotech China Co., Ltd., Shanghai, China) (primer concentrations were adjusted to 5–50 nmol/l). The final volume was adjusted to 20 μl with DEPC water. Following transfection, the PCR reaction solution used to detect the relative expression levels of miR-32 in GC cells (SGC-7901) included the following: 12.5 μl SYBR-Green/ROX qPCR master mix, 1.5 μl forward and reverse primers (including primers for miR-32 and U6) and 1 μl template DNA. The final volume was adjusted to 25 μl with DEPC water (each sample experiment was repeated four times). The following reaction conditions were used: uracil-DNA glycosylase pretreatment at 50°C for 2 min, initial denaturation at 95°C for 10 min, denaturation at 95°C for 15 sec and extension for 60 sec at 60°C for 40 cycles.

### Scratch-wound assay

Transiently transfected cells in logarithmic growth phase were seeded onto 6-well plates (5×10^5^/well) following culture for 24 h. At 24 h after seeding, the confluent cell monolayers were wounded with a pipette. Exfoliated cells were washed off using phosphate-buffered saline (PBS) and culture was continued in fresh medium without fetal bovine serum (FBS). Wound closure was monitored by microscopy at various times (0, 6 and 24 h). Visual fields (n=4) of each insert were randomly frozen under an inverted fluorescence microscope (IX71, Olympus, Tokyo, Japan). Migration activity was calculated as the mean distance between the edges of three points. Healing rate = (mean original distance - mean distance at a time point)/mean original distance × 100. Each test group was assayed in triplicate.

### Migration assay

Transiently transfected cells were trypsinized and suspended without serum-free RPMI-1640 culture medium. The suspended cells were seeded in the upper chamber of the Transwell^®^ insert and RPMI-1640 medium containing 20% FBS (600 μl) was added to the lower chamber of the Transwell insert. Following culturing at 37°C in a humidified 5% CO_2_ incubator for 24 h, the inserts were washed with PBS. A cotton swab was used to remove adherent cells on the inner side of the upper chamber membrane, then the chamber membranes were fixed in paraformaldehyde (4%) for 10 min. Coomassie Blue (600 μl) was added into each well and incubated at room temperature for 15 min. The inserts were washed again and the upper chamber was left to dry naturally. Visual fields (n=15) of each insert were randomly counted under an upright light microscope (BX51, Olympus) and the average value was calculated.

### Cell proliferation assay by cell counting kit-8 analysis

The cells were digested, resuspended and the cell concentration was adjusted (5,000/well). Next, the cells were seeded onto a 96-well plate and cultured at 37°C in a humidified 5% CO_2_ incubator until the cells had adhered. The cells were transiently transfected using Lipofectamine 2000, changing the medium after 4 h. CCK-8 solution (10 μl) was added to each well of the plate at 3 time points (24, 48 and 72 h after medium replacement) and the cells continued to be incubated on the plate. The absorbance was measured at 450 nm using a multifunctional microplate reader at 3 time points (1, 2 and 4 h after incubation). Cell growth curves were determined using the value of absorbance (at 450 nm) at various time points. Finally, the cell growth inhibition rate (IR) was calculated using the following formula: IR = (1 − A_experimental_/A_control_) × 100, where A represents the absorbance value.

### Statistical analysis

All experiments were repeated at least three times. Statistical analysis was performed using SPSS 19.0 (SPSS, Inc., Chicago, IL, USA). Data are presented as mean ± standard deviation and groups were compared using one-way analysis of variance. P<0.05 was considered to indicate a statistically significant difference.

## Results

### Expression of miR-32 in SGC-7901 cells transfected with miR-32-mimic and -inhibitor

**Figure 1:**
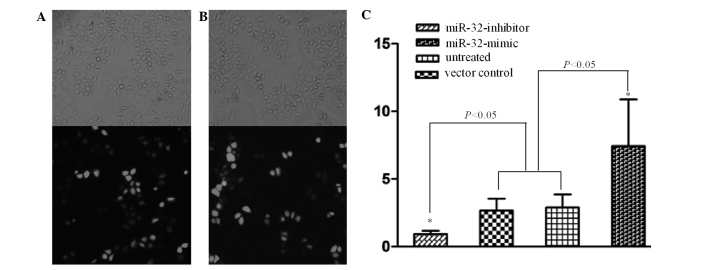
Transfection efficiency was observed under an inverted microscope following transfection with miR-32 (A) -mimic and (B) -inhibitor at 48 h. (A and B) Results showed that >80% of cells were labeled with green fluorescence. (C) Relative expression of miR-32 in SGC-7901 cells at 24 h post-transfection measured by qPCR. The results showed that miR-32 expression was significantly upregulated in the miR-32-mimic group and significantly downregulated in the miR-32-inhibitor group. ^*^P<0.05. qPCR, quantitative real-time PCR; miR-32, microRNA-32.

The transfection efficiency was confirmed under the inverted microscope. The results showed that >80% cells were labeled with GFP and SGC-7901 cells had been successfully transfected with miR-32-mimic and -inhibitor (Fig. 1A and B). Compared with the control group, the expression of miR-32 in the miR-32-mimic group was significantly downregulated and in the miR-32-inhibitor group significantly upregulated (P<0.05; Fig. 1C).

### miR-32 inhibits the migration and invasion of SGC-7901 cells in vitro

**Figure 2:**
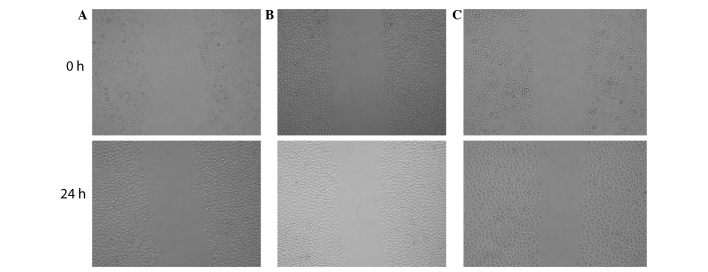
Cell migration ability assay of SGC-7901 cells by scratch-wound model. Confluent cell monolayers were wounded with a pipette tip. Wound closure was monitored by microscopy (magnification, ×200) following 24 h. The results showed that the migration ability of the miR-32-mimic group was significantly lower than that of the other groups (P<0.05). (A) miR-32-mimic, (B) untreated and (C) miR-32-inhibitor groups. miR-32, microRNA-32.

**Figure 3:**
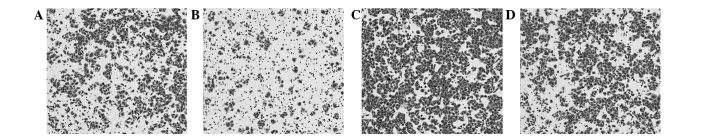
Cell invasion ability of SGC-7901 cells by Transwell^®^ assay. Visual fields (n=15) of each insert were randomly counted under a microscope (magnification, ×200). The results showed that the invasion cell number in the miR-32-mimic group was significantly less than that of the other groups (P<0.05). (A) miR-32-inhibitor, (B) miR-32-mimic, (C) untreated and (D) empty vector control groups. miR-32, microRNA-32.

The scratch-wound assay was used to measure and compare the scratch breadth at 24 h following transfection. The results showed that the migration ability of the miR-32-mimic group (61.39±2.21 vs. 64.42±2.15%; healing rate, 4.71±1.66%) was significantly lower than the the untreated group (27.49±2.15 vs. 60.4±0.73%; healing rate, 55.97±2.95%) and inhibitor (29.97±0.66 vs. 64.86±0.36%; healing rate, 53.79±0.76%) groups (Fig. 2). Furthermore, the Transwell assay was used to detect the cell invasion ability. The number of cells that had traversed the membrane were counted following transfection for 48 h. Compared with the control group, the miR-32-mimic group significantly reduced the invasion ability of GC cells. By contrast, the miR-32-inhibitor group significantly increased the invasion ability (P<0.05) (Table I; Fig. 3).

### miR-32 inhibits cell proliferation

To investigate the effect of miR-32 on the growth of human GC, the SGC-7901 cell line was transfected with miR-32-mimic and -inhibitor and a control vector for 48 and 72 h. Cell viability was performed by the CCK-8 assay. The results showed that high expression of miR-32 in the miR-32-mimic group significantly inhibited cell proliferation compared with the miR-32-inhibitor, control and untransfected groups (P<0.05; Table II).

## Discussion

According to the Lauren classification, there are two major types of GC: Intestinal and diffuse. The diffuse type is associated with the mutation, deletion and promoter methylation of E-cadherin (10). While, based on the Correa hypothesis of gastric carcinogenesis, the intestinal type of gastric carcinogenesis is a multistage process with the following order of gastric carcinogenesis: Normal gastric mucosa, superficial gastritis, atrophic gastritis, intestinal metaplasia, intraepithelial neoplasia and carcinoma. A number of molecules and complex regulatory networks are involved in gastric carcinogenesis, including infection with *Helicobacter pylori*, activation of oncogenes, inactivation of cancer suppressor genes and changes in epigenetic modification (11). Gastric carcinogenesis is an important issue which remains to be solved by clinical scientists. Critical molecular mechanisms and biomarkers for GC must be found to improve the identification of high risk warning signs, early diagnosis, prognosis and effective therapeutic options.

Caudal type homeobox transcription factor 2 (CDX2), is comprised of 311 residues and binds to corresponding DNA sequences through a helix-loop-helix domain. CDX2 is expressed in intestinal epithelial, pancreas ductal and acinar epithelial cells which originate from the endoderm, but is absent in the esophagus and normal gastric mucosa epithelial cells. CDX2 is an intestine-specific nuclear transcription factor and is important in regulating the proliferation and differentiation of normal intestinal epithelial cells (12).

It has been previously shown that ectopic expression of CDX2 causes changes associated with gastric intestinal metaplasia and GC (13–17). Etopic expression of CDX2 in gastric epithelial cells leads to the genesis of intestinal metaplasia, followed by intestinal GC. *Helicobacter pylori* infection causes the chronic inflammation of gastric mucosa which may progress to intestinal metaplasia. Intestinal metaplasia epithelial cells are well recognized as precancerous lesions and increase the risk of intestinal GC. CDX2 is found in almost 100% of gastric intestinal metaplasia tissue and the majority of early GC, particularly intestinal GC (14,15). Our previous study (17,18) also showed that CDX2 mRNA is absent in normal gastric tissue, but ectopic expression presents in intestinal metaplasia, gastric epithelial dysplasia and GC. In addition, the positive rate of CDX2 in intestinal GC was significantly higher than in the diffuse type. A previous study (15) reported that CDX2 locates, not only in intestinal metaplasia, but also in incomplete intestinal metaplasia. However, the expression levels in incomplete intestinal metaplasia were much lower. Consistently, CDX2 expression in the goblet and columnar cells of the incomplete intestinal metaplasia tissue was much lower than in intestinal metaplasia. Although the structure of incomplete intestinal metaplasia cells is similar to colonic epithelial cells, they express much lower levels of CDX2. From intestinal to dysplasia metaplasia and then GC, the expression of CDX2 decreases. The aforementioned studies illustrate that CDX2 functions as a cancer suppressor gene in gastric carcinogenesis, as well as colon cancer. The low expression of CDX2 in intestinal and dysplasia metaplasia is a critical marker of high risk gastric carcinogenesis. Simultaneously with the progression of malignant lesions, the expression of CDX2 in GC tissue declines; the expression of CDX2 in early GC is significantly higher than in advanced GC and low status lymphatic and distant metastasis GC. Following surgical treatment, patients with CDX2-positive expression demonstrated a higher survival rate compared with those with CDX2-negative expression (19). In addition, the *in vitro* experiments showed that GC cells transfected with CDX2 exhibited typical apoptotic morphological changes (20). Overexpression of CDX2 arrests GC cells in the G0/G1 stage of the cell cycle and induces cell apoptosis. All the results showed that CDX2 is involved in the regulation of GC proliferation and metastasis (21). In conclusion, CDX2 is a cancer suppressor gene of GC.

miRNAs are important for post-transcriptional gene regulation via binding to target mRNA to mediate destabilization and translational inhibition. It has been previously confirmed that miRNAs are involved in cell proliferation, differentiation and apoptosis. Therefore, miRNAs have been found to closely correlate with the genesis and progression of tumors. Previous studies have identified that specific miRNA have the function to suppress cancer, while others, by contrast, promote cancer. miRNA, as a newly identified gene regulation factor, is involved in the complex control network of the cell and mediates a dispensable regulation model of gene expression (4).

It remains unclear whether CDX2, as a nuclear transcription regulator, directly upregulates or downregulates miRNA to mediate GC cell proliferation and metastasis. Our previous studies showed that SGC-7901 GC cells transfected with CDX2 exhibit changes in biological behavior. The miRCURY LNA™ array was used to screen for the altered expression of miRNA in SGC-7901 cells transfected with CDX2. It was found that the expression of 59 miRNAs was changed significantly in the transfected group (transfected with pEGFP-N1-CDX2). Of the 59 miRNAs, 25 were upregulated and 34 were downregulated by more than double. miR-32 and miR-374a, which were found to be upregulated significantly, were randomly selected to confirm the result of the miRNA expression chip by qPCR. The qPCR results were consistent with the results from the chip (unpublished data). Following the overexpression of miR-32 at 48 and 72 h, cell proliferation and invasion ability were examined and found to be markedly decreased. By contrast, the suppressed expression of miR-32 led to a marked increase in cell proliferation and invasion ability. The target prediction software miRanda, TargetScan and miRtarget were used to predict miR-32 targets and SMAD7, GATA6, MYH9 and SOX4 were identified. SMAD7 is a suppressive member of the SMAD family and is involved in the transforming growth factor-β/SMAD signaling pathway and GATA6 and SOX4 are key molecules in the Wnt signaling pathway. Therefore, CDX2 may mediate miR-32 to achieve its anticancer effect. As a result, miR-32 was selected in the present study as the target gene to further study its effect on the biological behavior of GC cells.

miR-32 eukaryotic expression vectors (mimic and inhibitor) were successfully constructed and SGC-7901 GC cells were transfected with these vectors. miR-32 expression was upregulated and downregulated by various methods and dynamic biological changes were observed in the GC cells. The results showed that, compared with the inhibitor and control groups (blank and empty vector controls), the miR-32-mimic group inhibited cell proliferation and migration at 48 and 72 h following transfection (P<0.05). Therefore, miR-32 markedly inhibits the malignant behavior of SGC-7901 cells. However, the mechanisms by which CDX2 mediates the miR-32-altered phenotype of GC cells *in vivo* and *in vitro,* and which molecules miR-32 interacts with to regulate the phenotype of GC cells, remain to be solved in future studies.

In conclusion, the present study confirmed that miR-32 notably impacts the biological behavior of GC cells and the upregulation of miR-32 markedly inhibits the proliferation and migration of GC cells. These results are likely to contribute to the identification of the molecular mechanisms of CDX2 antigastric growth and metastasis and the development of targeted therapeutics for GC.

## Figures and Tables

**Table I:** Cell invasion ability for GC cells by Transwell^®^ assay.

Variable	Visions, n	Invading cells, n
miR-32-mimic group	15	45.93±4.63^a^
miR-32-inhibitor group	15	76.12±3.62
Empty vector group	15	82.19±3.32
Untreated group	15	93.93±7.09

a P<0.05, vs. miR-32-inhibitor, untreated and empty vector groups.

Data are expressed as mean ± SD of triplicate experiments determined by a count of cells/vision. GC, gastric cancer; miR-32, microRNA-32.

**Table II:** Cell viability measured by cell counting kit-8 assay at various times.

	24 h		48 h		72 h	
			
Variable	A450 value	IR, %	A450 value	IR, %	A450 value	IR, %
miR-32-mimic group	0.41±0.16	36.46±14.33	0.58±0.12^a^	43.474±18.63	0.65±0.10^a^	45.05±23.76
miR-32-inhibitor group	0.36±0.06	38.54±13.45	0.78±0.08	36.300±14.10	1.13±0.09	24.81±2.85
Empty vector group	0.37±0.07	31.50±11.08	0.83±0.15	33.580±86.69	1.22±0.29	35.42±23.82
Untreated group	0.43±0.19	0	0.97±0.19	0	1.13±0.67	0

a P<0.05, vs. miR-32-inhibitor, empty vector and untreated groups.

Data are expressed as mean ± standard deviation of triplicate experiments. IR, inhibition rate; miR-32, microRNA-32.

## References

[1] Compare D, Rocco A, Nardone G (2010). Risk factors in gastric cancer. Eur Rev Med Pharmacol Sci.

[2] Jemal A, Siegel R, Xu J, Ward E (2010). Cancer statistics 2010. CA Cancer J Clin.

[3] Petrocca F, Visone R, Onelli MR, Shah MH, Nicoloso MS, de Martino I, Iliopoulos D, Pilozzi E, Liu CG, Negrini M (2008). E2F1-regulated microRNAs impair TGFbeta-dependent cell-cycle arrest and apoptosis in gastric cancer. Cancer Cell.

[4] Lagos-Quintana M, Rauhut R, Lendeckel W, Tuschl T (2001). Identification of novel genes coding for small expressed RNAs. Science.

[5] Lau NC, Lim LP, Weinstein EG, Bartel DP (2001). An abundant class of tiny RNAs with probable regulatory roles in *Caenorhabditis elegans*. Science.

[6] Lee RC, Ambros V (2001). An extensive class of small RNAs in *Caenorhabditis elegans*. Science.

[7] Bueno MJ, Pérez de Castro I, Malumbres M (2008). Control of cell proliferation pathways by microRNAs. Cell Cycle.

[8] Cho WC (2007). OncomiRs: the discovery and progress of microRNAs in cancers. Mol Cancer.

[9] Bartel DP (2004). MicroRNAs: genomics, biogenesis, mechanism and function. Cell.

[10] Lauren P (1965). The two histological main types of gastric carcinoma: diffuse and so-called intestinal-type carcinoma. An attempt at a histo-clinical classification. Acta Pathol Microbiol Scand.

[11] Tamura G, Yin J, Wang S, Fleisher AS, Zou T, Abraham JM, Kong D, Smolinski KN, Wilson KT, James SP (2000). E-Cadherin gene promoter hypermethylation in primary human gastric carcinomas. J Natl Cancer Inst.

[12] Yuasa Y (2003). Control of gut differentiation and intestinal-type gastric carcinogenesis. Nat Rev Cancer.

[13] Bonhomme C, Duluc I, Martin E, Chawengsaksophak K, Chenard MP, Kedinger M, Beck F, Freund JN, Domon-Dell C (2003). The Cdx2 homeobox gene has a tumour suppressor function in the distal colon in addition to a homeotic role during gut development. Gut.

[14] Bai YQ, Yamamoto H, Akiyama Y, Tanaka H, Takizawa T, Koike M, Kenji Yagi O, Saitoh K, Takeshita K, Iwai T, Yuasa Y (2002). Ectopic expression of homeodomain protein CDX2 in intestinal metaplasia and carcinomas of the stomach. Cancer Lett.

[15] Eda A, Osawa H, Yanaka I, Satoh K, Mutoh H, Kihira K, Sugano K (2002). Expression of homeobox gene CDX2 precedes that of CDX1 during the progression of intestinal metaplasia. J Gastroenterol.

[16] Mutoh H, Sakurai S, Satoh K, Tamada K, Kita H, Osawa H, Tomiyama T, Sato Y, Yamamoto H, Isoda N (2004). Development of gastric carcinoma from intestinal metaplasia in Cdx2-transgenic mice. Cancer Res.

[17] Mao ZB, Zhang JF, Xu Z, Zhu HJ, Zhang JG, Pan ZP, Xiao F, Yang JL (2009). Ectopic expression of guanylyl cyclase C in gastric cancer as a potential biomarker and therapeutic target. J Dig Dis.

[18] Zhang JF, Zhang JG, Kuai XL, Zhang H, Jiang W, Ding WF, Li ZL, Zhu HJ, Mao ZB (2013). Reactivation of the homeotic tumor suppressor gene CDX2 by 5-aza-2′-deoxycytidine-induced demethylation inhibits cell proliferation and induces caspase-independent apoptosis in gastric cancer cells. Exp Ther Med.

[19] Qin R, Wang NN, Chu J, Wang X (2012). Expression and significance of homeodomain protein Cdx2 in gastric carcinoma and precancerous lesions. World J Gastroenterol.

[20] Xie Y, Li L, Wang X, Qin Y, Qian Q, Yuan X, Xiao Q (2010). Overexpression of Cdx2 inhibits progression of gastric cancer in vitro. Int J Oncol.

[21] Barros R, Camilo V, Pereira B, Freund JN, David L, Almeida R (2010). Pathophysiology of intestinal metaplasia of the stomach: emphasis on CDX2 regulation. Biochem Soc Trans.

